# A checklist and areography of the longhorn beetles (Coleoptera, Cerambycidae) of Pirin Mountains, Bulgaria

**DOI:** 10.3897/BDJ.10.e93718

**Published:** 2022-10-31

**Authors:** Georgi Georgiev, Vladimir Sakalian, Plamen Mirchev, Margarita Georgieva, Sevdalin Belilov

**Affiliations:** 1 Forest Research Institute, Bulgarian Academy of Sciences, Sofia, Bulgaria Forest Research Institute, Bulgarian Academy of Sciences Sofia Bulgaria; 2 Institute of Biodiversity and Ecosystem Research, Bulgarian Academy of Sciences, Sofia, Bulgaria Institute of Biodiversity and Ecosystem Research, Bulgarian Academy of Sciences Sofia Bulgaria

**Keywords:** Pirin Mountains, cerambycids, faunistics, chorology

## Abstract

**Background:**

The longhorn beetles fauna of Pirin Mountains, Bulgaria, was studied, based on literature data and original material. As a result, 100 taxa from five subfamilies are listed for the area, as follows: Prioninae (7 taxa), Lepturinae (31 taxa), Spondylidinae (9 taxa), Cerambycinae (28 taxa) and Lamiinae (25 taxa).

**New information:**

This study presents two new records for Pirin Mts. (*Oxymiruscursor* and *Tetropiumfuscumfuscum*) and new localities or additional information for 13 cerambycid taxa (species and subspecies). The 100 longhorn beetle taxa belong to 17 zoogeographical categories and eight complexes. The European complex occupies a dominant position (34%), followed by those from Palaearctic (17%), Eurosiberian (15%), Mediterranean (15%), European-Iranoturanian (9%), Balkan endemic (5%) and Holarctic (4%) complexes.

## Introduction

Pirin is the second highest mountain in Bulgaria. The highest peak of the mountain, Vihren (2914 m), occupies the third position on the Balkan Peninsula after Musala (2925 m) in Rila (Bulgaria) and Mytikas (2918 m) in Olympus (Greece). The average altitude of Pirin Mts. is 1033 m a.s.l. and the total area is 2585 km^2^.

The vegetation of Pirin Mts. is vertically divided into three altitude belts: forest, subalpine and alpine. The lower part of the forest belt is predominantly made up of broad-leaved species stands (*Carpinusbetulus*, *Quercuspetraea*, *Fagussylvatica*, *Populustremula* etc.) and the upper part is mainly occupied by conifers (*Pinusnigra*, *P.sylvestris*, *P.peuce*, *P.heldreichii*, *P.mugo*, *Abiesalba* and *Piceaabies*) (Stoyanov 1966).

The Bulgarian cerambycid fauna (Coleoptera, Cerambycidae) is relatively well studied. Fragmentary data about findings of longhorn beetles in different mountains of the country, including Pirin, are available in many publications (Heyrovský 1931, Kantardjiewa-Minkova 1932, Kantardjiewa-Minkova 1934, Minkova 1957, Minkova 1961, Angelov 1967, Ganev 1984, Ganev 1985, Ganev 1986, Angelov 1995, Doychev and Georgiev 2004, Georgiev et al. 2005, Migliaccio et al. 2007, Rapuzzi and Georgiev 2007, etc.). Recently, fragmentary data have been summarised in separate lists for some Bulgarian mountains: Eastern Rhodopes (Georgiev et al. 2004), Western Rhodopes (Georgiev et al. 2006), West Balkan Range (Georgiev 2011), Vitosha (Topalov 2018), Strandzha (Georgiev et al. 2018), Belasitsa (Georgiev et al. 2019) and Rila (Georgiev et al. 2021). However, there is no checklist of cerambycid fauna of Pirin Mountains.

The aim of this study is to summarise the published data about longhorn beetles in the Pirin Mts., report new records and provide a chorological analysis of the cerambycid fauna of this mountain range.

## Materials and methods

The longhorn beetles of Pirin Mts. were studied using literature data, our original records and unpublished materials from the entomological collection of the National Museum of Natural History, Sofia. The original material was collected on flowers, host plants and using an interception trap in a tree crown of *Pinusheldreichii*.

In this study, we followed the classification and nomenclature of longhorn beetles proposed by Sama (2002), Sama (2013), Biscaccianti (2007), Lobl and Smetana (2010), Miroshnikov (2016), Miroshnikov (2021), Zamoroka et al. (2022) and Danilevsky (2022), without indication of tribes and subgenera.

The zoogeographical characterisation of longhorn beetles was made on the basis of chorotypes (Vigna-Taglianti et al. 1999) and recent distribution of the taxa (Danilevsky 2022). According to Georgiev and Hubenov (2006) and Sakalian and Langourov (2007) conceptions, the established taxa are arranged in 18 chorotypes (areographic categories).

This paper provides a map with all known localities of the longhorn beetles recorded from Pirin Mts. It includes new localities from the current study and those already published. The records without a specific location, such as "Pirin Mt.", were not marked on the map.

Thecerambycid specimens collected in this study were deposited in the private entomological collection of Georgi Georgiev (mentioned with the abbreviation [GG]).

## Checklists

### Checklist

#### Ergates faber faber

(Linnaeus, 1760)

5DA871F4-E158-5C40-8177-A0E2C86E2413

##### Materials

- **Type status:** Other material. **Occurrence:** recordedBy: G. Georgiev leg. [GG]; sex: 1 female; occurrenceID: 1F526295-081D-561F-BDE3-4224D3344C7B; **Location:** country: Bulgaria; locality: Sandanski; **Event:** verbatimEventDate: 13/07/2010

##### Distribution

West Palaearctic subspecies (Danilevsky 2022).

#### Mesoprionus besikanus

(Fairmaire, 1855)

BC0BAE2C-E754-5F6F-97A3-43CC11B4A976

##### Materials

- **Type status:** Other material. **Occurrence:** recordedBy: G. Georgiev leg. [GG]; sex: 1 male; occurrenceID: 49971BE7-1E39-5A82-99ED-39D191D8CD72; **Location:** country: Bulgaria; locality: Peyo Yavorov hut; verbatimLocality: tree trap in *Pinusheldreichii* stand; verbatimElevation: 1886 m a.s.l.; verbatimLatitude: 41.825139; verbatimLongitude: 23.375639; **Event:** startDayOfYear: 03/08/2020; endDayOfYear: 30/10/2020

##### Distribution

East Mediterranean species (Danilevsky 2022).

#### Carilia virginea virginea

(Linnaeus, 1758)

6FE60E8B-6DB4-50CE-9F3B-77AE03B89ECD

##### Materials

- **Type status:** Other material. **Occurrence:** recordedBy: G. Georgiev leg. [GG]; sex: 2 males, 1 female; occurrenceID: D9552788-FD98-51B6-B16F-AE57FA7CFAD0; **Location:** country: Bulgaria; locality: Peyo Yavorov hut; verbatimElevation: 1750 m a.s.l.; **Event:** verbatimEventDate: 03/08/2020

##### Distribution

West Eurosiberian species (Danilevsky 2022).

#### Oxymirus cursor

(Linnaeus, 1758)

6A1D96D8-2503-54E1-BA9B-83069E0E2FEC

##### Materials

- **Type status:** Other material. **Occurrence:** recordedBy: G. Georgiev leg. [GG]; sex: 1 male; occurrenceID: A4716EEA-1076-5646-9EB9-969B76134037; **Location:** country: Bulgaria; locality: Above Bansko; **Event:** verbatimEventDate: 17/06/2006

##### Distribution

West Eurosiberian species (Danilevsky 2022).

#### Pachytodes erraticus

(Dalman, 1817)

66356D2D-4D78-509D-9AFE-8D4C42FD676D

##### Materials

- **Type status:** Other material. **Occurrence:** recordedBy: N. Simov leg. [GG]; sex: 1 female; occurrenceID: 81A397FA-9725-5CF5-B4B7-C200E5E68367; **Location:** country: Bulgaria; locality: Kalimantsi vill.; **Event:** verbatimEventDate: 06/06/2006

##### Distribution

European-Iranian species (Danilevsky 2022).

#### Pedostrangalia verticalis

Germar, 1822

AA588F89-5589-5D05-A61F-27DC21E308E7

##### Materials

- **Type status:** Other material. **Occurrence:** recordedBy: G. Georgiev leg. [GG]; sex: 1 male, 1 female; occurrenceID: 0FE1B1A0-C0DB-503A-9D14-0D076DCE9DDE; **Location:** country: Bulgaria; locality: Peyo Yavorov hut; verbatimElevation: 1750 m a.s.l.; verbatimLatitude: 41.824056; verbatimLongitude: 23.378472; **Event:** verbatimEventDate: 03/08/2020

##### Distribution

Northeast Mediterranean species (Danilevsky 2022).

#### Pseudovadonia livida livida

(Fabricius, 1777)

1EB21344-53B3-54B3-BC4D-FA2B9C1A38F6

##### Materials

- **Type status:** Other material. **Occurrence:** recordedBy: N. Simov leg. [GG]; sex: 2 males; occurrenceID: 198F0B2A-AD84-5830-B29D-A74D95D5C9BD; **Location:** country: Bulgaria; locality: Kalimantsi vill.; **Event:** verbatimEventDate: 06/06/2006

##### Distribution

European subspecies (Danilevsky 2022).

#### Rutpela maculata maculata

(Poda von Neuhaus, 1761)

22DA44C2-731D-5199-9C46-5570B7513603

##### Materials

- **Type status:** Other material. **Occurrence:** recordedBy: G. Georgiev leg. [GG]; sex: 1 male; occurrenceID: DFC61675-6F84-5C03-9A40-C41BC6B29417; **Location:** country: Bulgaria; locality: Peyo Yavorov hut; verbatimElevation: 1750 m a.s.l.; verbatimLatitude: 41.824056; verbatimLongitude: 23.378472; **Event:** verbatimEventDate: 03/08/2020

##### Distribution

European-Anatolian subspecies (Danilevsky 2022).

#### Rutpela nigra nigra

(Linnaeus, 1758)

4A8CEED5-AA5A-5C24-866D-94ADD89C9E4D

##### Materials

- **Type status:** Other material. **Occurrence:** recordedBy: N. Simov leg. [GG]; sex: 1 female; occurrenceID: A35478D3-4611-542B-A9F5-364627A7E98B; **Location:** country: Bulgaria; locality: Koprivlen vill.; verbatimElevation: 600 m a.s.l.; **Event:** verbatimEventDate: 06/06/2006
- **Type status:** Other material. **Occurrence:** recordedBy: N. Simov leg. [GG]; sex: 1 female; occurrenceID: DEB958C5-8C51-5E08-A813-BC5922B35805; **Location:** country: Bulgaria; locality: Gorno Spantchevtsi vill.; verbatimElevation: 600 m a.s.l.; **Event:** verbatimEventDate: 06/06/2006

##### Distribution

European-Anatolian subspecies (Danilevsky 2022).

#### Stictoleptura scutellata scutellata

(Fabricius, 1781)

816D005E-5A52-5520-8C55-B767AC4EFB76

##### Materials

- **Type status:** Other material. **Occurrence:** recordedBy: G. Georgiev leg. [GG]; sex: 1 female; occurrenceID: BDAAD8B2-BBB0-57E3-8EF9-F56100B594B5; **Location:** country: Bulgaria; locality: Peyo Yavorov hut; verbatimElevation: 1750 m a.s.l.; verbatimLatitude: 41.824056; verbatimLongitude: 23.378472; **Event:** verbatimEventDate: 03/08/2020

##### Distribution

European subspecies (Danilevsky 2022).

#### Tetropium fuscum fuscum

(Fabricius, 1787)

9A2B8414-42E2-5F0E-82EA-4A85BB71A8B4

##### Materials

- **Type status:** Other material. **Occurrence:** recordedBy: G. Georgiev leg. [GG]; sex: 1 male; occurrenceID: 1CC6778F-5724-5186-874C-1315BDEA4920; **Location:** country: Bulgaria; locality: Bansko; verbatimElevation: 1180 m a.s.l.; verbatimLatitude: 41.809944; verbatimLongitude: 23.468361; **Event:** verbatimEventDate: 08/06/2019

##### Distribution

Transholarctic species (Danilevsky 2022).

#### Callimus angulatus angulatus

(Schrank, 1789)

90292267-8A91-50DE-9EA0-33AA46810E6C

##### Materials

- **Type status:** Other material. **Occurrence:** recordedBy: N. Simov leg. [GG]; sex: 1 male; occurrenceID: 91AE5D50-EF4F-5251-8CD6-A58CD70CE605; **Location:** country: Bulgaria; locality: Koprivlen vill.; verbatimElevation: 600 m a.s.l.; **Event:** verbatimEventDate: 06/06/2006

##### Distribution

Euromediterranean subspecies (Danilevsky 2022).

#### Clytus rhamni rhamni

Germar, 1817

D10CC22F-7AD0-598B-8FDA-14A6594951B4

##### Materials

- **Type status:** Other material. **Occurrence:** recordedBy: D. Chobanov leg. [GG]; sex: 2 males; occurrenceID: A88ED3F7-C766-5689-8AE8-A2A411B111A6; **Location:** country: Bulgaria; locality: Kalimantsi vill.; verbatimLocality: Shirokata Burchina place; verbatimElevation: 350 m a.s.l.; **Event:** verbatimEventDate: 01/06/2002
- **Type status:** Other material. **Occurrence:** recordedBy: N. Simov leg. [GG]; sex: 1 male, 1 female; occurrenceID: D554C836-6D28-5408-85D1-12DAE85B6A61; **Location:** country: Bulgaria; locality: Kalimantsi vill.; **Event:** verbatimEventDate: 06/06/2006

##### Distribution

Northeast Mediterranean subspecies (Danilevsky 2022).

#### Agapanthia kirbyi kirbyi

(Gyllenhal, 1817)

3A82BAA1-C98B-5618-9383-C901449CCEB0

##### Materials

- **Type status:** Other material. **Occurrence:** recordedBy: G. Georgiev leg. [GG]; sex: 1 female; occurrenceID: 46747924-CCCA-5F0C-A06C-58ABD5E728A1; **Location:** country: Bulgaria; locality: Oshtava vill.; **Event:** verbatimEventDate: 15/06/2008

##### Distribution

European-Iranian subspecies (Danilevsky 2022).

#### Phytoecia coerulescens coerulescens

(Scopoli, 1763)

71BA1C32-DAF7-507A-A61E-0C9A069755D1

##### Materials

- **Type status:** Other material. **Occurrence:** recordedBy: N. Simov leg. [GG]; sex: 1 male; occurrenceID: E619F1FC-A2B1-5A87-BC9E-245749131A90; **Location:** country: Bulgaria; locality: Kalimantsi vill.; **Event:** verbatimEventDate: 06/06/2006

##### Distribution

West Palaearctic subspecies (Danilevsky 2022).

## Analysis

**Figure 1.**
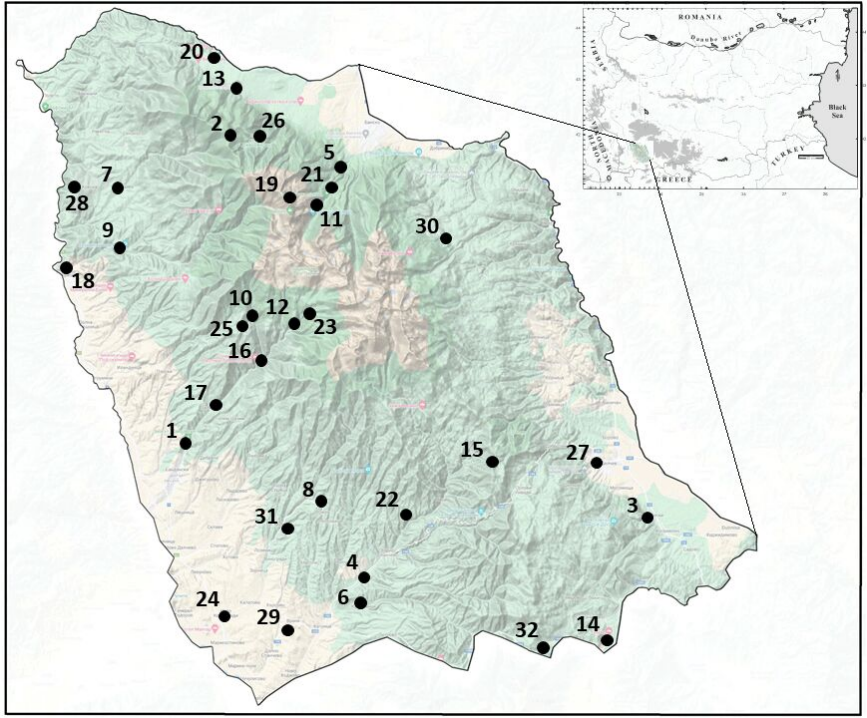
Localities of the collected cerambycids in Pirin Mts.: 1 – Sandanski; 2 – Peyo Yavorov hut; 3 – Koprivlen; 4 – Gorno Spantchevtsi; 5 – Bansko; 6 – Kalimantsi; 7 – Oshtava; 8 – Rozhen Monsarety; 9 – Vlahi; 10 – Tremoshnitsa River; 11 – Banderitsa hut; 12 – Popina laka; 13 – Kulinoto; 14 – Nova Lovcha; 15 – Oreliak; 16 – Sandanska Bistritsa River; 17 – Lilyanovo; 18 – Kresna; 19 – Vihren; 20 – Predela; 21 – Demyanitsa River; 22 – Pirin; 23 – Yane Sandanski hut; 24 – Ilindentsi; 25 – Eltepe; 26 – Bayuvi Dupki; 27 – Gotse Delchev; 28 – Stara Kresna; 29 – Katuntsi; 30 – Gotse Delchev hut; 31 – Melnik; 32 – Paril

According to the present study, 15 species are reported for Pirin Mountains. Of these, two species *Oxymiruscursor* and *Tetropiumfuscumfuscum* are new records and new localities or new specimens are given for 13 species. Summarising our data with the published one, 100 cerambycid taxa from 32 localities are recorded in Pirin Mts. (Fig. 1).

The recorded cerambycid taxa belong to five subfamilies: Prioninae (7 taxa), Lepturinae (31 taxa), Spondylidinae (9 taxa), Cerambycinae (28 taxa) and Lamiinae (25 taxa) (Table 1).

The established cerambycid taxa belong to 17 areographical categories separated in eight complexes (Table 2).

**Figure 2.**
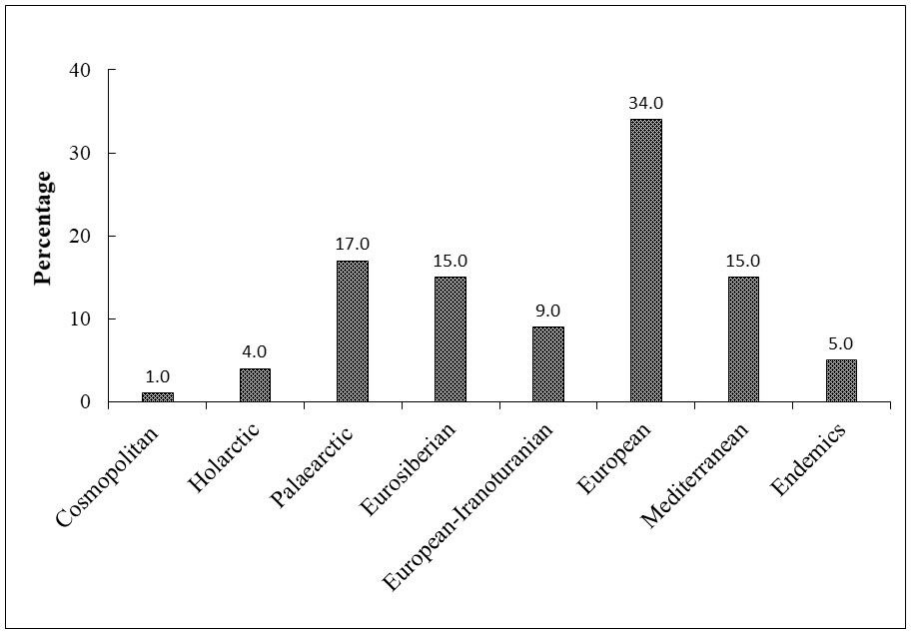
Arealographic complexes of the longhorn beetles in Pirin Mts.

The taxa from the European complex are dominant in Pirin Mts. (34%) followed by those from Palaearctic (17%), Eurosiberian (15%) and Mediterranean (15%) complexes (Fig. 2).

## Discussion

The number of cerambycid taxa found in Pirin Mts. (100 species and subspecies) is closest to the West Balkan Range (107 taxa) (Georgiev 2011, Gradinarov and Petrova 2019), Belasitsa Mt. (110 taxa) (Georgiev et al. 2019), Vitosha Mt. (122 taxa) (Topalov 2018) and Rila Mt. (126 taxa) (Georgiev et al. 2021). It is also comparable to the number of cerambycids in other Bulgarian mountains studied: Strandzha (154 taxa) (Georgiev et al. 2018) and Western Rhodopes (161 taxa) (Georgiev et al. 2006).

In this study, taxa of the European complex occupy a dominant position (34%). They are connected with deciduous forests, which cover the largest parts of the lower territories of Pirin Mts. The second place is taken by the species and subspecies belonging to the Palaearctic complex (17%). These more eurybiont taxa are normally better represented in the high mountains, because of the harsh climatic conditions. Eurosiberian and Mediterranean complexes with equal value (15%) occupy the third place. This pattern differentiates Pirin from the rest of the studied high mountains - Vitosha (Topalov 2018) and Rila (Georgiev et al. 2021), where the third place is taken by Eurosiberian taxa. The difference is due not only to the more southerly location of the Pirin Mts., but also to the presence of the large valleys of Struma and Mesta Rivers, which allows the penetration of Mediterranean taxa.

The high territories mostly covered by coniferous trees and shrubs are favourable for distribution of the Eurosiberian taxa (fourth place). The refugial character of the region is underlined by the presence of five (5.0%) Balkan endemic cerambycids.

Concerning the other two studied mountains in Bulgaria - Strandzha (Georgiev et al. 2018) and Belasitsa (Georgiev et al. 2019), domination of European cerambycids were also established (33.1% and 38.2%, respectively), but the Mediterranean taxa have a greater share in both mountains (27.3% and 19.1%, respectively). In addition, European-Iranoturanian taxa are mostly represented in Strandzha Mt. (13.6%) compared to other mountains (7.2–11.1%). The level of Balkan and Bulgarian endemics is higher in Belasitsa Mt. – 9 taxa (8.2%), followed by Pirin 5 taxa (5.1 %), Rila Mt. – 5 (4.0%), Strandzha Mt. – 5 (3.3%) and Vitosha Mt. – 2 taxa (1.7%). Evidently, the conditions in Belasitsa Mt. and especially the distribution of relict forests of *Castaneasativa* is the most suitable for occurrence of endemics there.

In future investigations, the cerambycid fauna of Pirin Mt. will undoubtedly be enriched with species trophically associated with coniferous trees in the the mountains of Bulgaria, such as: *Lepturoboscavirens* (Linnaeus, 1758), *Acmaeopsseptentrionis* Thomson, 1866, *Acmaeopspratensis* (Laicharting, 1784), *Evodinellusclathratus* (Fabricius, 1793), *Molorchusmarmottanimarmottani* Brisout de Barneville, 1863, *Pachytalamed* (Linnaeus, 1758), *Paracorymbiamaculicornis* (De Geer, 1775), *Pidonialurida* (Fabricius, 1793), *Semanotusundatus* (Linnaeus, 1758), *Acanthocinusgriseus* (Fabricius, 1793), *Acanthocinusreticulatus* Razoumowsky, 1789 and *Monochamussartor* (Fabricius, 1787) (Georgiev and Hubenov 2006).

It is expected that longhorn beetles with Eurosiberian, Palearctic and European distribution associated with coniferous, broadleaved and grass vegetation in other mountains in south-western Bulgaria – Rila, Western Rhodopes and Belasitsa (Georgiev et al. 2006, Georgiev et al. 2019, Georgiev et al. 2021) will be also recorded: *Prionuscoriarius* (Linnaeus, 1758), *Anoploderasexguttata* (Fabricius, 1775), *Anoploderarufipesrufipes* (Schaller, 1783), *Dinopteracollaris* (Linnaeus, 1758), *Rhagiummordax* (DeGeer, 1775), *Rhagiumsycophanta* (Schrank, 1781), *Strangaliaattenuata* (Linnaeus, 1758), *Necydalismajor* Linnaeus, 1758, *Clytuslama* Mulsant, 1847, *Ropalopusungaricusinsubricus* (Germar, 1823), *Stenocorusmeridianus* (Linnaeus, 1758), *Aromiamoschatamoschata* (Linnaeus, 1758), *Callidiumviolaceum* (Linnaeus, 1758), *Callidiumaeneumaeneum* (De Deer, 1775), *Callidiumcoriaceum* Paykull, 1800, *Plagionotusarcuatusarcuatus* (Linnaeus, 1758), *Pyrrhidiumsanguineum* (Linnaeus, 1758), *Xylotrechusrusticus* (Linnaeus, 1758), *Agapanthiadahlidahli* (C. F. W. Richter, 1820), *Dorcadionfulvumerythropterum* Fischer von Waldheim, 1823, *Dorcadiontauricumtauricum* Waltl, 1838, *Lamiatextor* (Linnaeus, 1758), *Leiopuslinnei* Wallin, Nylander & Kvamme, 2009, *Obereaerythrocephalaerythrocephala* (Schrank, 1776), *Phytoeciacylindrica* (Linnaeus, 1758), *Phytoeciaicterica* (Schaller, 1783), *Phytoecianigricornis* (Fabricius, 1782), *Phytoeciavirgulavirgula* (Charpentier, 1825), *Pogonocherushispidulus* (Piller & Mitterpacher, 1783), *Saperdacarcharias* (Linnaeus, 1758) and *Tetropspraeustuspraeustus* (Linnaeus, 1758).

Due to the specific location of Pirin in south-eastern Europe and the penetration of Mediterranean influence along the valleys of the Struma and Mesta Rivers, it is very likely that species with European-Iranian and Mediterranean distribution reported for Rila and Belasitsa (Georgiev et al. 2019, Georgiev et al. 2021) may also be reported, for example: *Grammopteraabdominalis* (Stephens, 1831), *Xylosteusspinolae* Frivaldszky von Frivald, 1837, *Cerambyxnodulosusnodulosus* Germar, 1817, *Lioderinalinearis* (Hampe, 1871), *Molorchusumbellatarumumbellatarum* (Schreber, 1759), Ropalopus clavipes (Fabricius, 1775), *Agapanthiacynaraecynarae* (Germar, 1817), *Mesosacurculionoides* (Linnaeus, 1760) and *Stenideageneigenei* (Aragona, 1830).

Regarding the endemic complex, in future research, it is likely that other species reported for neighbouring Rila and Belasitsa Mts. may also be found: *Agapanthiaschurmanni* Sama, 1979, *Dorcadionaethiopsstrumense* Danilevsky, 2014, *Dorcadionaxillare* Küster, 1847, *Dorcadionsturmii* Frivaldszky von Frivald, 1837 and *Phytoeciageniculataorientalis* Kraatz, 1871 (Georgiev et al. 2019, Georgiev et al. 2021).

Only three cerambycids were recorded in trophic associations with tree and shrub species in the Pirin Mts.: *Arhopalusrusticusrusticus* and *Pogonocherusfasciculatusfasciculatus* were reared from stems and branches of *Pinusnigra* (Doychev et al. 2017) and *Phymatodesglabratus* from a stem of *Cupressussempervirens* (Doychev and Georgiev 2006).

In conclusion, finding of 100 taxa (approximately 35% of longhorn beetles in Bulgaria) indicates that this taxonomic group is not yet well-studied and about 70–80 taxa are expected to be recorded in future investigations in the Pirin Mts.

## Supplementary Material

XML Treatment for Ergates faber faber

XML Treatment for Mesoprionus besikanus

XML Treatment for Carilia virginea virginea

XML Treatment for Oxymirus cursor

XML Treatment for Pachytodes erraticus

XML Treatment for Pedostrangalia verticalis

XML Treatment for Pseudovadonia livida livida

XML Treatment for Rutpela maculata maculata

XML Treatment for Rutpela nigra nigra

XML Treatment for Stictoleptura scutellata scutellata

XML Treatment for Tetropium fuscum fuscum

XML Treatment for Callimus angulatus angulatus

XML Treatment for Clytus rhamni rhamni

XML Treatment for Agapanthia kirbyi kirbyi

XML Treatment for Phytoecia coerulescens coerulescens

## Figures and Tables

**Table 1.** Table 1. Localities and chorotypes of longhorn beetles (Coleoptera: Cerambycidae) in the Pirin Mts.

**N**	**Taxon**	**Locality (N – see Fig. 1)**	**Reference**	**Chorotype**
	**Subfamily Prioninae Latreille, 1802**			
1	*Aegosomascabricorne* (Scopoli, 1763)	Sandanski (1)	Minkova (1957), Ganev (1985), Ganev (1986), Rapuzzi and Georgiev (2007), Georgiev (2020)	European-Iranian
		Rozhen Monsarety (8) Kalimantsi vill. (6)	Gradinarov et al. (2020)	
2	*Prinobiusmyardislamorum* Danilevsky, 2012	Vlahi vill. (9)	Rapuzzi and Georgiev (2007), Migliaccio et al. (2007)	Pontomediterranean
3	*Rhaesusserricollis* Motschulsky, 1838	Tremoshtitsa River (10)	Ganev (1986)	East European-Iranian
4	*Tragosomadepsarium* (Linnaeus, 1767)	Banderitsa hut (11)	Dimitrov (1963)	Eurosiberian
5	*Mesoprionusbesikanus* (Fairmaire, 1855)	Tremoshtnitsa River (10)	Ganev (1986)	East Mediterranean
		Pirin Mt.	Angelov (1995)	
		Peyo Yavorov hut (2)	**New locality record**	
6	*Prionuscoriarius* (Linnaeus, 1758)	Bansko (5)	Ganev (1984)	West Palaearctic
		Popina laka (12), Tremoshtitsa River (10)	Ganev (1986)	
		Pirin Mt.	Angelov (1995)	
		Kulinoto loc., Razlog (13)	Georgiev (2020)	
		Paril vill.	Georgiev (2020) Gradinarov et al. (2020)	
7	*Ergatesfaberfaber* (Linnaeus, 1760)	Nova Lovcha vill. (14)	Gradinarov et al. (2020)	West Palaearctic
		Sandanski (1)	**New locality record**	
	**Subfamily Lepturinae Latreille, 1802**			
8	*Alosternatabacicolortabacicolor* (DeGeer, 1775)	Bansko (5), Kalimantsi vill. (6)	Rapuzzi and Georgiev (2007)	West Eurosiberian
		Kalimantsi vill. (6)	Gradinarov et al. (2020)	
9	*Anastrangaliadubiadubia* (Scopoli, 1763)	Banderitsa (11)	Heyrovský (1931), Kantardjiewa-Minkova (1932)	Euromediterranean
		Pirin Mt.	Angelov (1995)	
10	*Anastrangaliasanguinolenta* (Linnaeus, 1760)	Oreliak Reserve (15)	Georgiev et al. (2005)	West Eurosiberian
		Sandanska Bistritsa River (16)	Georgiev (2020)	
		Bansko (5)	Gradinarov et al. (2020)	
11	*Etorofuspubescens* (Fabricius, 1787)	Pirin Mt.	Bringmann (1996)	European-Anatolian
12	*Grammopteraruficornisruficornis* (Fabricius, 1781)	Kalimantsi vill. (6)	Gradinarov et al. (2020)	European-Anatolian
13	*Pachytodescerambyciformis* (Schrank, 1781)	Banderitsa hut (11)	Angelov (1967)	European
14	*Pachytodeserraticus* (Dalman, 1817)	Lilyanovo vill. (17), Kresna (18)	Angelov (1967)	European-Iranian
		Banderitsa Chalet - Vihren Peak (19)	Gradinarov et al. (2020)	
		Kalimantsi vill. (6)	**New locality record**	
15	*Lepturaaurulenta* Fabricius, 1793	Popina laka loc. (12)	Ganev (1986)	Euromediterranean
16	*Lepturaquadrifasciataquadrifasciata* Linnaeus, 1758	Bansko (5) Pirin Mt.	Heyrovský (1931), Kantardjiewa-Minkova (1932)	Transpalaearctic
17	*Paracorymbiafulva* (DeGeer, 1775)	Lilyanovo vill. (17), Kresna (18)	Angelov (1967)	European-Anatolian
		Bansko (5)	Gradinarov et al. (2020)	
18	*Paracorymbiapallens* (Brullé, 1832)	Vlahi vill. (9)	Gradinarov et al. (2020)	Northeast Mediterranean
19	*Pedostrangaliaverticalis* Germar, 1822	Kalimantsi vill. (6)	Rapuzzi and Georgiev (2007)	Northeast Mediterranean
		Vlahi vill. (9)	Gradinarov et al. (2020)	
		Peyo Yavorov hut (2)	**New locality record**	
20	*Pedostrangaliarevestita* (Linnaeus, 1767)	Sandanski (1)	Migliaccio et al. (2007)	European
21	*Pseudovadonialividalivida* (Fabricius, 1777)	Bansko (5)	Kantardjiewa-Minkova (1932)	European
		Lilyanovo vill. (17)	Angelov (1967)	
		Kalimantsi vill. (6)	**New locality record**	
22	*Rutpelamaculatamaculata* (Poda von Neuhaus, 1761)	Peyo Yavorov hut (2)	Georgiev (2020)	European-Anatolian
		Peyo Yavorov hut (2)	**Record in this study**	
23	*Rutpelanigranigra* (Linnaeus, 1758)	Kalimantsi vill. (6)	Gradinarov et al. (2020)	European-Anatolian
		Koprivlen vill. (6) Gorno Spantchevtsi vill. (4)	**New locality record** **New locality record**	
24	*Rutpelaseptempunctataseptempunctata* (Fabricius, 1793)	Bansko (5)	Kantardjiewa-Minkova (1932)	European
		Banderitsa Chalet - Vihren Peak (19)	Gradinarov et al. (2020)	
25	*Stenurellabifasciataintermedia* Holzschuh, 2006	Bansko (5)	Heyrovský (1931)	Balkan endemic
		Kresna gorge (18)	Danilevsky (2011)	
		Kalimantsi vill. (6)	Gradinarov et al. (2020)	
26	*Stenurellamelanuramelanura* (Linnaeus, 1758)	Kalimantsi vill. (6)	Rapuzzi and Georgiev (2007) Gradinarov et al. (2020)	Transpalaearctic
		Predela loc. (20)	Georgiev (2020)	
27	*Stictolepturacordigeracordigera* (Füsslins, 1775)	Popina laka (12)	Ganev (1986)	European-Iranian
28	*Stictolepturarubrarubra* (Linnaeus, 1758)	Demyanitsa River (21)	Kantardjiewa-Minkova (1932)	Eurosiberian
29	*Stictolepturascutellatascutellata* (Fabricius, 1781)	Popina laka (12)	Ganev (1986)	European
		Pirin vill. (22)	Gradinarov et al. (2020)	
		Peyo Yavorov hut (2)	**New locality record**	
30	*Vadoniadojranensismahri* Holzschuh, 1986	Kalimantsi vill. (6)	Minkova (1961)	Balkan endemic
31	*Oxymiruscursor* (Linnaeus, 1758)	Bansko (5)	**First record for Pirin Mts.**	West Eurosiberian
32	*Cariliavirgineavirginea* (Linnaeus, 1758)	Pirin Mt.	Kantardjiewa-Minkova (1932), Minkova (1957)	West Eurosiberian
		Peyo Yavorov hut (2)	**New locality record**	
33	*Cortoderafemorata* (Fabricius, 1787)	Bansko (5)	Rapuzzi and Georgiev (2007)	West Eurosiberian
34	*Cortoderahumeralishumeralis* (Schaller, 1783)	Kalimantsi vill. (6)	Rapuzzi and Georgiev (2007)	European-Anatolian
35	*Pachytaquadrimaculata* (Linnaeus, 1758)	Pirin Mt.	Angelov (1995) Migliaccio et al. (2007)	Transpalaearctic
		Tremoshtnitsa River (10)	Ganev (1986)	
36	*Rhagiumbifasciatum* Fabricius, 1775	Pirin Mt.	Kantardjiewa-Minkova (1932)	European-Iranian
		Yane Sandanski hut (23)	Angelov (1967)	
		Pirin vill. (22)	Gradinarov et al. (2020)	
37	*Rhagiuminquisitorinquisitor* (Linnaeus, 1758)	Yane Sandanski hut (23)	Angelov (1967)	Eurosiberian
		Popina laka (12)	Ganev (1986)	
		Ilindentsi vill. (24)	Doychev et al. (2019)	
		Eltepe shelter (25)	Georgiev (2020)	
		Bansko (5), Vichren peak (19)	Georgiev and Simov (2006)	
38	*Xylosteusbartoni* Obenberger & Mařan, 1933	Banderitsa hut (11), Vihren (19)	Georgiev and Simov (2006)	Balkan endemic
		Vihren (19), Bayuvi Dupki - Dzhindhiritsa Reserve (26)	Gradinarov et al. (2020)	
	**Subfamily Spondylidinae Audinet-Serville, 1832**					
39	*Alocerusmoesiacus* (Frivaldszky von Frivald, 1837)	Kalimantsi hut (6)	Gradinarov et al. (2020)	Transmediterranean
40	*Archopalusferus* (Mulsant, 1839)	Tremoshnitsa River (10)	Ganev (1986)	Transpalaearctic
		Pirin Mt.	Angelov (1995)	
41	*Archopalusrusticusrusticus* (Linnaeus, 1758)	Bansko (5)	Ganev (1984)	Transpalaearctic
		Tremoshnitsa River (10)	Ganev (1986)	
		Predela loc. (20)	Georgiev (2020)	
		Ilindentsi vill. (24), reared from *Pinusnigra*	Doychev et al. (2017)	
42	*Asemumstriatum* (Linnaeus, 1758)	Demjanitsa hut (21)	Angelov (1967)	Transholarctic
43	*Nothorhinapunctata* (Fabricius, 1798)	Yane Sandanski hut (23)	Migliaccio et al. (2007)	Transpalaearctic
44	*Tetropiumcastaneum* (Linnaeus, 1758)	Bansko (5)	Kantardjiewa-Minkova (1932)	Transpalaearctic
45	*Tetropiumfuscumfuscum* (Fabricius, 1787)	Bansko (5)	**First record for Pirin Mts.**	Transholarctic
46	*Saphanuspiceusganglbaueri* Brancsik, 1886	Pirin Mt.	Minkova (1957), Angelov (1995)	European
		Kalimantsi vill. (6)	Rapuzzi and Georgiev (2007)	
		Peyo Yavorov hut (2)	Georgiev (2020)	
47	*Spondylisbuprestoides* (Linnaeus, 1758)	Pirin Mt.	Kantardjiewa-Minkova (1932)	Transpalaearctic
	**Subfamily Cerambycinae Latreille, 1802**			
48	*Icosiumtomentosumatticum* Ganglbauer, 1882	Kalimantsi vill. (6)	Gradinarov and Sivilov (2020)	Northeast Mediterranean
49	*Anaglyptusmysticus* (Linnaeus, 1758)	Gotse Delchev (27)	Georgiev (2020)	European-Anatolian
50	*Phymatodesglabratus* (Charpentier, 1825)	Ilindentsi vill. (24), reared from *Cupressussempervirens*	Doychev and Georgiev (2006), Doychev et al. (2017)	European-Anatolian
51	*Phymatodeslividus* (Rossi, 1794)	Kalimantsi vill. (6)	Migliaccio et al. (2007), Rapuzzi and Georgiev (2007)	Euromediterranean
52	*Phymatodestestaceus* (Linnaeus, 1758)	Kalimantsi vill. (6)	Gradinarov et al. (2020)	Transholarctic
53	*Cerambyxmiles* Bonelli, 1812	Kalimantsi vill. (6)	Gradinarov et al. (2020)	European-Anatolian
54	*Cerambyxscopoliiscopolii* Fuessly, 1775	Bayuvi dupki loc. (26)	Georgiev (2020)	European-Anatolian
55	*Chlorophorusfiguratus* (Scopoli, 1763)	Pirin Mt.	Rapuzzi and Georgiev (2007)	West Eurosiberian
		Kalimantsi vill. (6)	Gradinarov et al. (2020)	
56	*Chlorophorusherbstii* (Brahm, 1790)	Bansko (5)	Angelov (1967)	Eurosiberian
57	*Chlorophorusvariusvarius* (O. F. Müller, 1766)	Sandanski (1)	Angelov (1967)	West Eurosiberian
		Tremoshnitsa River (10)	Ganev (1986)	
58	*Chlorophorussartor* (O. F. Müller, 1766)	Gotse Delchev (27)	Georgiev (2020)	West Palaearctic
		Banderitsa loc. (11), Sheitan Dere River, Stara Kresna vill. (28)	Gradinarov et al. (2020)	
59	*Clytusrhamnirhamni* Germar, 1817	Gotse Delchev (27)	Georgiev (2020)	Northeast Mediterranean
		Kalimantsi vill. (6)	**New locality record**	
60	*Echinocerusfloralisaulicus* (Laicharting, 1784)	Sandanski (1)	Angelov (1967)	Transpalaearctic
		Bayuvi Dupki loc. (26)	Georgiev (2020)	
61	*Neoplagionotusbobelayeibobelayei* (Brullé, 1832)	Vlahi vill. (9)	Ganev (1985)	East European-Iranian
62	*Xylotrechusarvicolaarvicola* (Olivier, 1795)	Predela (20)	Georgiev (2020)	Euromediterranean
63	*Xylotrechusstebbingi* Gahan, 1906	Lilyanovo vill. (17)	Gradinarov and Sivilov (2020)	Subcosmopolitan
64	*Rosalia alpina* (Linnaeus, 1758)	Banderitsa hut (11)	Angelov (1967)	European-Anatolian
		Popina laka loc. (12)	Ganev (1986)	
65	*Axinopalpisgracilisgracilis* (Krynicki, 1832)	Kalimantsi vill. (6), Ilindentsi vill. (24), Lilyanovo vill. (17)	Gradinarov et al. (2020)	European-Iranian
66	*Stromatiumauratum* (Böber, 1793)	Sandanski (1)	Kovacs et al. (1998)	Transmediterranean
67	*Molorchusminorminor* (Linnaeus, 1758)	Bansko (5)	Georgiev et al. (2005)	Transpalaearctic
		Vihren peak (19)	Gradinarov et al. (2020)	
68	*Obriumbrunneum* (Fabricius, 1793)	Bansko (5)	Angelov (1988)	European-Anatolian
		Pirin Mt.	Angelov (1995)	
		Banderitsa chalet (11)	Gradinarov et al. (2020)	
69	*Stenhomalusbicolorbicolor* (Kraatz, 1862)	Pirin Mt.	Heyrovský (1931)	Northeast Mediterranean
70	*Purpuricenusbudensis* (Götz, 1783)	Pirin vill. (22), Stara Kresna vill. (28)	Georgiev (2020)	West Palaearctic
71	*Purpuricenuskaehlerirossicus* Danilevsky, 2019	Popina laka (12)	Ganev (1986)	East European
		Gotse Delchev – Katuntsi (29)	Gradinarov et al. (2020)	
72	Callimusangulatusangulatus (Schrank, 1789)	Koprivlen vill. (3)	Rapuzzi and Georgiev (2007)	Euromediterranean
		Kalimantsi vill. (6), Pirin vill. (22)	Gradinarov et al. (2020)	
		Koprivlen vill. (3)	**Record in this study**	
73	*Callimusfemoratus* (Germar, 1824)	Katuntsi vill. (29)	Gradinarov et al. (2020)	East European-Iranian
74	*Stenopterusflavicornis* Küster, 1846	Vlahi vill. (9)	Ganev (1985)	Northeast Mediterranean
		Gotse Delchev (27)	Georgiev (2020)	
75	*Stenopterusrufusrufus* (Linnaeus, 1767)	Gotse Delchev (27)	Georgiev (2020)	European
	**Subfamily Lamiinae Latreille, 1825**			
76	*Acanthocinusaedilis* (Linnaeus, 1758)	Banderitsa hut (11)	Dimitrov (1963)	Transpalaearctic
		Sandanski (1)	Angelov (1967)	
		Gotse Delchev hut (30)	Rapuzzi and Georgiev (2007)	
77	*Aegomorphusclavipes* (Schrank, 1781)	Pirin Mt.	Rapuzzi and Georgiev (2007)	European
		Kalimantsi vill. (6)	Gradinarov et al. (2020)	
78	*Aegomorphuskrueperi* (Kraatz, 1859)	Kulinoto loc. near Razlog (13)	Georgiev (2020)	Balkan endemic
79	*Agapanthiacardui* (Linnaeus, 1767)	Yane Sandanski hut (23)	Angelov (1967)	European
80	*Agapanthiavillosoviridescens* (De Geer, 1775)	Yane Sandanski hut (23)	Angelov (1967)	Eurosiberian
81	*Agapanthiaviolacea* (Fabricius, 1775)	Pirin Mt.	Bringmann (1995)	European-Anatolian
		Melnik (31)	Georgiev (2020)	
82	*Agapanthiakirbyikirbyi* (Gyllenhal, 1817)	Gotse Delchev hut (30)	Georgiev (2020)	European-Iranian
		Kalimantsi vill. (6)	Gradinarov et al. (2020)	
		Oshtava vill. (7)	**New locality record**	
83	*Anaesthetistestaceatestacea* (Fabricius, 1781)	Gotse Delchev (27), Predela loc. (20)	Georgiev (2020)	European-Anatolian
		Paril vill. (32)	Gradinarov et al. (2020)	
84	*Dorcadionseptemlineatumseptemlineatum* Waltl, 1838	Sandanski (1)	Rapuzzi and Georgiev (2007)	Balkan endemic
85	*Neodorcadionbilineatum* (Germar, 1823)	Bansko (5), Kalimantsi vill. (6)	Gradinarov et al. (2020)	Northeast Mediterranean
86	*Exocentrusadspersus* Mulsant, 1846	Pirin Mt.	Heyrovský (1931), Kantardjiewa-Minkova (1934)	European-Anatolian
		Lilyanovo vill. (17)	Gradinarov et al. (2020)	
87	*Exocentruspunctipennispunctipennis* Mulsant & Guillebeau, 1856	Kalimantsi vill. (6)	Gradinarov et al. (2020)	European
88	*Morimusasperfunereus* Mulsant, 1862	Pirin Mt.	Bringmann (1997)	Northeast Mediterranean
		Bayuvi Dupki loc. (26)	Georgiev (2020)	
89	*Monochamusgalloprovincialispistor* (Germar, 1818)	Banderitsa hut (11)	Dimitrov (1963)	West Eurosiberian
		Pirin Mt.	Rapuzzi and Georgiev (2007)	
90	*Monochamussutorsutor* (Linnaeus, 1758)	Peyo Yavorov hut (2)	Rapuzzi and Georgiev (2007)	West Eurosiberian
		Pirin Mt.	Georgiev (2020)	
91	*Phytoeciaaffinisaffinis* (Harrer, 1784)	Yane Sandanski hut (23)	Bringmann (1998)	European-Anatolian
92	*Phytoeciacoerulescenscoerulescens* (Scopoli, 1763)	Kalimantsi vill. (6)	Rapuzzi and Georgiev (2007)	West Palaearctic
		Sandanska Bistritsa River (16)	Georgiev (2020)	
		Banderitsa Chalet - Vihren Peak (19)	Gradinarov et al. (2020)	
		Kalimantsi vill. (6)	**Record in this study**	
93	*Phytoeciavittipennisvittipennis* Reiche, 1877	Melnik (31)	Rapuzzi and Georgiev (2007)	Northeast Mediterranean
94	*Phytoeciaalbovittigera* Heyden, 1863	Vlahi vill. (9)	Gradinarov et al. (2020)	Northeast Mediterranean
95	*Phytoeciahirsutulahirsutula* (Frölich, 1793)	Kalimantsi vill. (6)	Gradinarov et al. (2020)	West Eurosiberian
96	*Pogonocherusfasciculatusfasciculatus* DeGeer, 1775	Sandanski, Melnik (31)	Bringmann and Döring (2001)	Transpalaearctic
		Banderitsa hut (11)	Doychev and Georgiev (2004)	
		Ilindentsi vill. (24), reared from *Pinusnigra*	Doychev et al. (2017)	
97	*Pogonocherusperroudiperroudi* Mulsant, 1839	Rhozhen Monastery (8)	Bringmann and Döring (2001), Migliaccio et al. (2007)	Transmediterranean
98	*Saperdapopulnea* (Linnaeus, 1758)	Sandanski (1)	Angelov (1967)	Transholarctic
99	*Saperdapunctata* (Linnaeus, 1767)	Lilyanovo vill. (17)	Gradinarov et al. (2020)	Euromediterranean
100	*Saperdascalarisscalaris* (Linnaeus, 1758)	Pirin vill.	Gradinarov et al. (2020)	Euromediterranean

**Table 2.** Areogeographic characterisation of cerambycids in Pirin Mts.

**Areographic categories and complexes**	**Number**	**Percentage**
**Cosmopolitan complex**	**1**	**1.0**
Subcosmopolitan	1	1.0
**Holarctic complex**	**4**	**4.0**
Transholarctic	4	4.0
**Palaearctic complex**	**17**	**17.0**
Transpalaearctic	12	12.0
West Palaearctic	5	5.0
**Eurosiberian complex**	**15**	**15.0**
Eurosiberian	5	5.0
West Eurosiberian	10	10.0
**European-Iranoturanian complex**	**9**	**9.0**
European-Iranian	6	6.0
East European-Iranian	3	3.0
**European complex**	**34**	**34.0**
Euromediterranean	7	7.0
European-Anatolian	16	16.0
European	10	10.0
East European	1	1.0
**Mediterranean complex**	**15**	**15.0**
Transmediterranean	3	3.0
East Mediterranean	1	1.0
Northeast Mediterranean	10	10.0
Pontomediterranean	1	1.0
**Balkan endemic complex**	**5**	**5.0**
Balkan endemics	5	5.0
**Total**	**100**	**100.0**
